# Biological roles of cysteine proteinases in the pathogenesis of *Trichomonas vaginalis*

**DOI:** 10.1051/parasite/2014054

**Published:** 2014-10-28

**Authors:** Hilda M. Hernández, Ricardo Marcet, Jorge Sarracent

**Affiliations:** 1 Parasitology Department, “Pedro Kourí” Tropical Medicine Institute Havana 10400 Cuba

**Keywords:** *Trichomonas vaginalis*, Trichomonosis, Cysteine proteinases, Pathogenesis

## Abstract

Human trichomonosis, infection with *Trichomonas vaginalis*, is the most common non-viral sexually transmitted disease in the world. The host-parasite interaction and pathophysiological processes of trichomonosis remain incompletely understood. This review focuses on the advancements reached in the area of the pathogenesis of *T. vaginalis*, especially in the role of the cysteine proteinases. It highlights various approaches made in this field and lists a group of trichomonad cysteine proteinases involved in diverse processes such as invasion of the mucous layer, cytoadherence, cytotoxicity, cytoskeleton disruption of red blood cells, hemolysis, and evasion of the host immune response. A better understanding of the biological roles of cysteine proteinases in the pathogenesis of this parasite could be used in the identification of new chemotherapeutic targets. An additional advantage could be the development of a vaccine in order to reduce transmission of *T. vaginalis*.

## Abstract (fr)

La trichomonose humaine, infection par *Trichomonas vaginalis*, est la maladie sexuellement transmissible non-virale la plus courante dans le monde. L’interaction hôte-parasite et les processus physiopathologiques de la trichomonose restent mal compris. Cette revue se concentre sur les progrès obtenus dans le domaine de la pathogenèse de *T. vaginalis*, spécialement le rôle des cystéine-protéases. Elle met en lumière les différentes approches dans ce domaine et donne la liste d’un groupe de cystéine-protéases de *T. vaginalis* impliquées dans des processus divers tels que l’invasion de la couche de mucus, la cytoadhérence, la cytotoxicité, la disruption du cytosquelette des globules rouges, l’hémolyse et l’échappement envers la réponse immunitaire de l’hôte. Une meilleure compréhension des rôles biologiques des cystéine-protéinases dans la pathogenèse de ce parasite pourrait être utile pour l’identification de nouvelles cibles chimiothérapeutiques. Un avantage supplémentaire pourrait être le développement d’un vaccin en vue de réduire la transmission de *T. vaginalis*.

## Introduction

Cysteine proteinases (CPs) from a variety of parasites such as *Plasmodium falciparum* [42], *Trypanosoma cruzi* [38], *Entamoeba histolytica* [79], *Leishmania (Viannia) braziliensis* [82], and *Trichomonas vaginalis* [81] have been characterized at molecular and cellular levels, and the function that proteinases play in these organisms is coming into focus [56]. Important roles have been proposed for CPs in diverse processes such as cytotoxicity, cytoadherence, metabolism, host cell invasion, molecule degradation, virulence factors, hemolysis, and host immune response evasion, among others [75, 89].

*Trichomonas vaginalis* is a parasitic protozoan that causes human trichomonosis, a sexually transmitted disease. This parasite is a major cause of vaginitis, cervicitis, urethritis, and prostatitis [17, 69]. The consequences for women with trichomonosis include enhanced risk for human immunodeficiency virus transmission [53], cervical cancer [1], and adverse pregnancy outcomes, which suggest a need for increased control efforts [88].

*Trichomonas vaginalis* infection is very complex, and the broad ranges of clinical symptoms are unlikely to be attributed to a single pathogenic process [86]. The exact mechanisms of the pathogenesis have not been clearly elucidated to date [92]. However, the sequencing of the *T. vaginalis* genome has led to knowledge of new gene families involved in the host pathogenesis, leading to new research to understand the mechanism of the parasite’s pathogenicity better [19]. Trichomonal cytoadherence to epithelial cells is a critical step in the initiation phase of the infection and subsequent pathogenesis [31]. This process is species-specific and capable of inducing gene upregulation not only in the parasite [57] but also in the host cell [58].

*Trichomonas vaginalis* possesses high levels of proteolytic activity, mainly of the CP type. Interestingly, up to 23 spots with proteolytic activity between 23 and 110 kDa have been detected using two-dimensional (2-D) substrate gel electrophoresis (zymograms) [74]. Additionally, Leon-Sicairos et al. demonstrated that more spots with proteolytic activity can be observed on the zymograms depending on the parasite’s *in vitro* growth conditions, especially iron concentration [67]. However, most of these spots are encoded by only nine distinct genes [80]. Currently, this parasite is estimated to contain in the order of 156 cysteine peptidases [51].

Despite the studies related to the trichomonad proteinases, only a few CPs have been identified and characterized. Nevertheless, the roles of some of them in the onset of the infection have been demonstrated [8, 13, 45, 70]. The parasite’s cysteine proteolytic activity is necessary for recognition and adhesion of the parasite to the epithelial cells of the host [51].

In this review, we examine the advances in the understanding of the importance of CPs in the pathogenesis exerted by *T. vaginalis*. There are numerous events in the parasite’s development where the contribution of CPs has been hinted at, but the enzymes involved have not been elucidated. Studies are now underway to characterize the specific roles of the trichomonad CPs in the pathogenesis better. The scope of this review encompasses biological processes where the involvement of these enzymes in the pathogenesis has been suggested.

## Pathogenesis

Trichomonad CPs are found in different cell compartments, i.e., lysosomes and plasma membranes, or even released into the culture medium through the lysosome and late/endosomal pathways [87]. *In vivo*, trichomonad CPs have been found in the vaginal secretions of patients with acute trichomonosis [39, 50, 70, 81, 98], and some of them are immunogenic [4, 5]. Some CPs have been involved in virulence mechanisms (Table 1).

**Table 1.** Virulence mechanisms that involve cysteine proteinases in *Trichomonas vaginalis*.

Virulence mechanisms	References
Invasion of the mucous layer	Lehker & Sweeney (1999) [65]
Cytoadherence	Arroyo & Alderete (1989, 1995); Mendoza-López et al. (2000); Hernández et al. (2004) [12, 13, 49, 70]
Cytotoxicity	Alvarez-Sánchez et al. (2000, 2007, 2008); Hernández-Gutíerrez et al. (2003, 2004); Kummer et al. (2008); de Jesus et al. (2009); Ramón-Luing et al. (2011) [8–10, 28, 45, 46, 60, 81]
Cytoskeleton disruption of red blood cells and hemolysis	Dailey et al. (1990); Fiori et al. (1993, 1997) [27, 36, 37]
Degradation of immunoglobulins	Provenzano & Alderete (1995); Hernández-Gutierrez et al. (2004) [45, 78]
Apoptosis	Chang et al. (2004, 2006); Sommer et al. (2005); Kang et al. (2006); Fichorova (2009) [24, 25, 32, 55, 91]

### Invasion of the mucous layer

The mucous layer of the genital tract is the first host surface encountered by trichomonads. Mucin, the major proteinaceous constituent of mucous, forms a lattice structure that serves as a formidable physical barrier to microbial invasion. Binding the parasite to mucin followed by its proteolytic degradation by mucinase appears to be the major mechanism by which *T. vaginalis* can gain access to the underlying epithelium. Five mucinases of identical molecular weight were found in trichomonad lysates and supernatants. These mucinases are cysteine-like peptidases [65].

Given that the urogenital region of women is a constantly changing environment, it is conceivable that interactions of trichomonads with mucin and/or vaginal epithelial cells fluctuate [5] and persist in a non-self-limiting fashion [43]. For example, hormones influence the exfoliation of the squamous vaginal epithelial cells and, in addition, the trichomonad cysteine proteinase released into the vaginal milieu [5] may contribute to desquamation of the vaginal and cervical epithelial tissue [98]. This local erosion permits the parasites access to extracellular matrix-basement membrane components, such as fibronectin, lamimin binding, *α*-actinin, enolase, and phosphoglucomutase, that in parallel plays a role in cytoadherence [3, 59].

### Cytoadherence

The adherence mechanism to mucin may allow trichomonads to gain a temporary foothold before penetration into the mucous layer and ultimate parasitism of the underlying epithelial cells. Adherence to host surfaces has been shown to be an early and critical step in *Trichomonas* pathogenesis [14]. Five trichomonad surface proteins, named adhesins (AP23, AP33, AP51, AP65, and AP120) [6, 14, 41, 63, 72], mediate adherence and these molecules are upregulated during attachment to vaginal epithelial cells [15, 41]. During this event, *T. vaginalis* perturbs the junctional complex in epithelial cells, producing a decrease in the transepithelial electrical resistance, alteration in the pattern of functional complex protein distribution, as was observed for E-cadherin and ZO-1, and enlargement of the spaces between epithelial cells. These effects were dependent on the parasite’s virulence, the expression of adhesion proteins on the parasite’s surface and the iron concentration in the medium [26]. Iron plays a critical role in the host-parasite interaction and modulates the expression of virulence factors in this protozoan [94]. Fluorescence and immuno-cytochemical experiments showed that high-iron-grown organisms coexpressed adhesins on the surface and intracellularly, in contrast with low-iron-grown parasites [41]. In concordance, in iron-depleted parasites, enzymes involved in energetic metabolism, proteolysis and hydrogenosomal iron-sulfur (Fe-S) proteins were downregulated or even suppressed. Thus, the iron modulates the expression of proteins in the parasite [29]. In addition, Zn^2+^ also affects the protein profile of *T. vaginalis*. Some proteins were up- or down-regulated in the presence of Zn^2+^, while others showed no changes. *T. vaginalis* differentially expresses 27 proteins in the presence of Zn^2+^, which suggests that this parasite has the capability to adapt to different environments. These differences in protein expression profiles correlated with changes in some of its virulence properties, such as cytotoxicity [96].

Interestingly, an analysis by mass spectrometry indicated that the 48- and 63-kDa proteins of *T. vaginalis* had identity with two adhesins: AP51 and AP65, respectively. This confirms the existence of multifunctional proteins in *T. vaginalis*, and suggests that AP51 and AP65, besides serving as adhesion molecules, could also act as heme- and hemoglobin-binding proteins [11]. Moreover, it has been demonstrated that the adhesin AP120 showed homology with a hydrogenosomal enzyme, the pyruvate ferredoxin oxidoreductase encoded by the *pfoa* gene. This homology suggests that this novel adhesin induced by iron could also be an example of a moonlighting protein in *T*. *vaginalis* [72]. Thus, it will be interesting to identify other alternative functions for these trichomonad proteins.

An iron-responsive promoter and other regulatory elements in the 5′-UTR of the AP65-1 gene were identified as a mechanism for the positive transcriptional regulation of trichomonad genes by iron [76]. Recently, two IRE (iron-responsive elements)-like hairpin-loop structures in mRNAs of differentially iron-regulated TVCP4 and TVCP12 cysteine proteinases, as well as IRP (iron regulatory proteins)-like trichomonad proteins were identified in *T. vaginalis*. These data suggested the existence in this protozoan of a post-transcriptional iron regulatory mechanism by an IRE/IRP-like system [90].

One report indicates that the reduced amounts of putrescine by inhibition of ornithine decarboxylase (ODC) significantly increased *T. vaginalis* adherence to vaginal epithelial cells mediated by protein adhesions. However, surprisingly and unexpectedly, trichomonal contact-dependent cytotoxicity was absent [40]. Recently, this effect was demonstrated by cytotoxicity and cell-binding assays followed by zymograms, as well as Western blot and indirect immunofluorescence assays using a specific anti-CP65 antibody to detect CP65 [9]. Trichomonads grown in the presence of the ODC inhibitor, 1-4 diamino-2-butanone, had lower levels of cytotoxicity that corresponded with diminished CP65 proteolytic activity when compared with untreated organisms handled identically. It was reversed by addition of exogenous putrescine, showing a direct link between polyamine metabolism and expression of the cytotoxic CP65 proteinase in the involved trichomonal host cellular damage [9].

Furthermore, it was demonstrated that trichomonad proteinase activity appears to be necessary for cytoadherence [12]. The protease inhibitors *N-α-p*-tosyl-L-lysine-chloromethyl ketone HCl (TLCK) and leupeptin were found to significantly reduce parasite to cervical adenocarcinoma (HeLa) cells and vaginal epithelial cells [12]. Exposure of TLCK-treated microorganisms to other cysteine proteinases restored cytoadherence levels, indicating that proteinase action on the parasite’s surface is a prerequisite for host cell attachment [13]. The exact function or the precise step for trichomonad proteinase involvement during parasite recognition and binding to epithelial cell surfaces is not known [12]. It is conceivable that unmasking of adhesins by proteinases residing on the parasite’s surface is required for host cell recognition and binding. It is equally possible that adhesins on trichomonad membranes exist as precursor forms which must be activated by specific proteinase digestion [12].

Using a cell-binding assay, a TLCK-sensitive 30-kDa cysteine proteinase with high affinity for the surface of cervical adenocarcinoma (HeLa) cells was identified in *T. vaginalis* extracts [13, 70]. A specific anti-CP30 antibody reduced cytoadherence by up to 50%. In addition, it was demonstrated that patients with trichomonosis possess antibodies to CP30 in both sera samples and vaginal swabs [70]. More recently, CP30 activity was found in all the vaginal washes of symptomatic women and in 80% of the vaginal washes of asymptomatic women [98]. Probably, besides CP30, other factors such as CP65, CP39, and CP62 may also play a role in leading to symptomatic infection [98]. CP30 was also detected in all the fresh culture isolates from symptomatic and asymptomatic women. This proteinase may be an important virulence factor of the parasite as its expression has been found to be higher in isolates causing symptomatic infection [99].

Recently, a proteomic analysis of *T. vaginalis* protein extracts was performed by Ramón-Luing et al. [80]. Nine CPs were identified in the 30-kDa region (TVCP1, TVCP2, TVCP3, TVCP4, TVCP4-like, TVCP12, TVCPT, TVLEGU-1, and another legumain-like CP). By two-dimensional Western blot, four papain-like CPs (TVCP2, TVCP4, TVCP4-like, and TVCPT), and one legumain-like CP (TVLEGU-1) showed the major reactive spots to *T. vaginalis*-positive patient sera. These data show that some CPs could be potential biomarkers for serodiagnosis of trichomonosis [80]. Recently, Rendón-Gandarilla et al. showed that TVLEGU-1 is a surface proteinase upregulated by iron, with affinity for the surface of cervical adenocarcinoma (HeLa) cells, that plays a major role in trichomonal cytoadherence. Hence, TVLEGU-1 is a novel virulence factor of *T. vaginalis* that is also released in vaginal secretions during infection [83].

Cuervo et al. performed a small-scale comparative analysis of soluble protein expression between *T. vaginalis* isolates exhibiting low- and high-virulence phenotypes. These analyses identified both quantitative and qualitative differences in protein expression profiles, including a number of proteins involved in carbohydrate and energy metabolism, cytoskeletal structure, and proteolysis [22]. Further, de Jesus et al. identified eight CPs that were differentially expressed between high- and low-virulence phenotypes. Seven of the eight CPs identified belong to Clan CA, family C1, cathepsin L-like CP, and one belongs to Clan CD, family C13, asparaginyl endopeptidase-like CP. A BLAST analysis followed by CLUSTAL alignment of amino acid sequences of differentially expressed CPs showed identity or high homology to the previously described CP cDNA clones CP1, CP3, and CP4, and to a secreted CP fraction of 30 kDa involved in apoptosis of vaginal epithelial cells [28].

In a study performed by our group, it was shown that anti-CP62 monoclonal antibodies (4D8 and 1A8) decrease cytoadherence of *T. vaginalis* to the cervical adenocarcinoma (HeLa) cell monolayer. The injection of these monoclonal antibodies into BALB/c mice by the intraperitoneal route conferred different protection levels against a challenge with the parasite [49]. Moreover, no cytotoxic effect of the monoclonal antibodies against the parasite was detected by monitoring the lactate dehydrogenase release by *T. vaginalis* in response to different antibody concentrations. On the other hand, anti-CP62 monoclonal antibodies were unable to inhibit the CP activity of *T. vaginalis*. These results suggest that the epitopes recognized by these antibodies are important in *T. vaginalis* cytoadherence and that the secreted proteinase shares epitopes with some structure in the parasite’s surface that is necessary for cytoadherence [49]. Another specific anti-CP TVLEGU-1 antibody can decrease the cytoadherence by inhibition of CP activity [83].

Further studies showed that anti-CP62 monoclonal antibodies (4D8 and 1A8) react with a different protein epitope of a repetitive nature found on CP62 and this could explain the differences among them in the protection grade obtained in the challenge experiments [48]. In addition, the intranasal immunization of mice with CP62 combined with cholera toxin or CpG adjuvant induced high levels of a specific anti-CP62 antibody in serum and vaginal secretions, and conferred protection against *T. vaginalis* [47]. Recently, CP62 was detected in all the vaginal swabs from symptomatic and asymptomatic women screened. Significant amounts of antigens were detected in vaginal swabs from symptomatic when compared with asymptomatic women, indicating that CP62 could be a virulence factor [50]. It will be interesting to determine the functions of CP62, and the environmental conditions that modulate their expression and possible participation in *T. vaginalis* cytopathogenicity.

Besides CPs, other surface domains such as lipophosphoglycan are also responsible for adherence of trichomonads to human vaginal epithelial cells. *T. vaginalis* lipophosphoglycan triggers a selective upregulation of cytokines by human female reproductive tract epithelial cells which promotes the adhesion and transmigration of neutrophils across the endothelium, and the macrophage inflammatory protein 3*α*, which is a chemoattractant for immune cells and is essential for dendritic cell maturation [35]. Another study has demonstrated that *T. vaginalis* LPG mutants reduced adherence to human ectocervical epithelial cell lines [16].

### Cytotoxicity

Evidence suggests that *T. vaginalis* may produce molecules that are delivered to target cells and mediate cytotoxicity through damage of the plasma membrane [8]. A specific anti-CP65 antibody of *T. vaginalis* reduced cytotoxicity to the cervical adenocarcinoma (HeLa) cell monolayer by up to 64% [8]. This has also been demonstrated for the CP39 proteinase. Parasites preincubated with the specific antibody to CP39 proteinase exhibited a reduction in their ability to destroy the cervical adenocarcinoma (HeLa) cell monolayer but not in cytoadherence, in a concentration-dependent manner [46]. This proteinase has been suggested as a potential biomarker for trichomonosis [81].

Under iron-restricted conditions there is an increase in the levels of trichomonal cytotoxicity over the cervical adenocarcinoma (HeLa) cell monolayer due to an increase in the TVCP65 proteolytic activity [10]. Likewise, an increase in the secreted CPs from the 30-kDa region (TVCP2, TVCP3, TVCP4, and TVCPT) was also observed, favoring their ability to induce human vaginal epithelial programmed cell death [60]. By a semiquantitative reverse transcription-polymerase chain reaction using mRNA from parasites grown in different iron concentrations, differences in the expression of some of the CP genes were also observed; some of them showed more transcript in iron-restricted conditions (TVCP12 and TVCP65), others in iron-rich conditions (TVCP4). These data suggested that different proteinases with similar molecular weight but different pIs are differentially regulated by iron and participate in virulence properties, such as cytoadherence, cytotoxicity, induction of apoptosis, and other still unknown functions [94].

Recent studies indicated that pretreatment of parasites with the specific Clan CA papain-like CP inhibitor l-3-carboxy-2,3-trans-epoxypropionyl-leucylamido(4-guanidino) butane (E-64) drastically reduced the cytotoxic effect, suggesting that *T. vaginalis* papain-like CPs are the main factors involved in the cellular damage [28].

### Cytoskeleton disruption of red blood cells and hemolysis

*Trichomonas vaginalis* has evolved multiple mechanisms for acquiring iron from specific iron-binding (lactoferrin) and iron-containing (hemoglobin and cytochrome) proteins [61]. The iron, which is an important nutrient for *T. vaginalis*, may be obtained by hemoglobin degradation after the lysis of erythrocytes [64]. Identification of a 60-kDa CP of *T. vaginalis*, which is capable of degrading hemoglobin into heme and globin, supports the supposition that this parasite may use hemoglobin as a source of iron [71].

Metabolically active parasites are necessary for lysis of erythrocytes [36]. CP inhibitors greatly reduced erythrocyte lysis, which suggests that CPs may be a lytic factor involved in hemolysis [27]. The lysis of the erythrocytes appears to be mediated by protein receptors on the surfaces of erythrocytes and parasites. Empirical evidence from studies with human erythrocytes suggests that perforin-like proteins (possibly cysteine proteinase) may be involved [37]. Carlton et al. identified 12 genes (TVSaplip1 to TVSaplip 12) containing pore-forming domains. TVSaplips are similar to amoebapore proteins secreted by *Entamoeba histolytica* and are candidate trichopores that mediate a cytolytic effect [19].

The mechanism of pore formation has been extensively studied in *E. histolytica* [44]. Acid pH causes the protonation of the basic His 75 residue that in turn triggers amoebapore dimerization as a result of the interaction of histidine with a negatively charged residue. The interaction of the three amoebapore dimers leads to the formation of a hexameric ring-like structure with a hydrophobic external surface and a hydrophilic inner channel [66]. *T. vaginalis*-mediated hemolysis is also triggered by an acidic pH and several TVSaplip domains show a basic His or Lys residue in the same key position as His of amoebapores, suggesting a conserved pH-dependent mechanism driving oligomerization [51].

Hemolysis seems to occur in three steps: a specific ligand-receptor interaction allows the trichomonad to attach itself to the erythrocytes, followed by the release of perforin-like proteins which form pores in the erythrocyte membrane. Finally, *T. vaginalis* detaches itself from the cell and cell lysis occurs [37].

### Evasion of the host immune response

The numerous CPs synthesized by *T. vaginalis* contribute significantly to immune evasion. The parasite’s ability to evade the host immune system is an important aspect of the pathogenesis. Avoidance of complement is used by *T. vaginalis* to overcome the human immune system. *T. vaginalis* has the advantage of living in a niche in which little complement is present [6]. Nevertheless, iron upregulates the expression of CPs, which have been found to degrade the C3 portion of complement on the surface of the organism; this allows the organism to evade complement-mediated destruction [6]. However, the particular proteinases responsible for this function have not been identified yet.

In addition, *T. vaginalis* displays other ways of evading the immune system. Provenzano and Alderete reported that numerous CPs secreted by *T. vaginalis* degrade IgG, IgM, and IgA, which allows the organism to survive the antibody response. Degradation of the heavy chain of IgG and IgA was observed following incubation with lysates and culture supernatants of *T. vaginalis* [78]. Among the CPs, TVCP39 is one of the papain-like proteinases that correspond to a single proteolytic spot of 39 kDa and pI 4.5 in 2-D substrate gel electrophoresis. It degrades several extracellular matrix proteins (including fibronectin, different types of collagen, immunoglobulin G (IgG) and IgA) and hemoglobin [45].

Moreover, secretory leukocyte protease inhibitor (SLPI) is a factor protecting the mucosal surface of the vagina [62]. Again, trichomonad proteinases are able to degrade SLPI and render it non-functional. In symptomatic women, this anti-inflammatory mediator was lower, possibly due to digestion by *Trichomonas* cysteine proteases [2]. Interestingly, SLPIs have also been shown to prevent HIV transmission, thus trichomonad proteinases may be partly responsible for the observed increase in risk of HIV acquisition in women with trichomonosis [93]. Recently, Huppert et al. showed that in adolescents and young adult females a depressed secretory leukocyte protease inhibitor (SLPI) level is strongly associated with *T. vaginalis* infections in a manner dependent on parasite load [54]. Moreover, experimental studies have proven that SPLI production by vaginal and cervical epithelial cells decreased in response to purified *T. vaginalis* LPG [33] and thus the lower SLPI levels observed clinically may be due to LPG and not just cystein proteases [34].

Lactobacilli are responsible for maintaining the acidic pH of the vagina (normal vaginal pH) and are considered protective of normal vaginal flora. Hydrogen peroxide produced by lactobacilli readily neutralizes the CPs, showing the protective effect of lactobacilli normal flora [7]. However, both an increase in the vaginal pH and reduction of the flora have been reported in patients with trichomonosis. This may be caused by phagocytosis of lactobacilli, which would enable the parasite to survive in a more basic milieu, subverting this host protective effect [84].

In addition, the *T. vaginalis* cysteine proteases including CP30 induce apoptosis in vaginal epithelial cells [91] and in multiple mucosal immune cell types [32]. In T cells, macrophages and dendritic cells, *T. vaginalis* led to apoptosis and production of immunosuppressive cytokines (IL-10, TGF*β*) [25]. *T. vaginalis* proteins (adhesins and CP30) induce caspase-mediated apoptosis and immunosuppressive cytokine response [24]. *T. vaginalis*-induced apoptosis in neutrophils has been linked to caspase-3 activation and reduced expression of the anti-apoptotic protein myeloid cell leukemia sequence 1 (Mcl-1) [55], and in macrophages it has been linked to extracellular signal-regulated kinase activation [23]. *T. vaginalis* infection has been shown to activate toll-like receptors (TLR)-4 by inducing undefined substance(s) released in the vaginal secretions [101]. To date, trichomonad ligands for TLR4 have not been identified. *T. vaginalis* infections of the mucocutaneous barrier could upregulate toll-like receptor (TLR) 2, 4, and 9 gene expression via the p38 mitogen-activated protein kinase pathway in cervical adenocarcinoma (HeLa) cells [24]. However, TLR4 was not upregulated by *T. vaginalis* in non-cancer human female genital tract epithelial cells [34].

## Cysteine proteinases of *T. vaginalis*. Possible target for chemotherapy and vaccine candidates

More than 180 million people worldwide are infected annually by *T. vaginalis* [97]. Metronidazole has been the standard therapy for the treatment of trichomonosis [53]. Resistance to the drug has been reported both *in vitro* and clinically [30, 95], suggesting a need to develop sustainable control strategies such as vaccination and development of new anti-*Trichomonas* drugs for the control of this disease. Currently, there is little knowledge about *T. vaginalis* surface antigens, and hence the considerations for the development of a potential vaccine are limited [21]. Similarly, the development of alternative antimicrobial strategies targeting virulence factors or based on immunotherapeutic approaches [18] would also depend on detailed knowledge of the pathogen pathobiology and the host defense mechanisms.

During infection with *T. vaginalis*, immunity has been difficult to achieve *in vivo*, since in humans, repeated infections with the parasite do not confer immune protection [52]. Despite this, antibodies have been found in the serum [98] and vaginal secretions of infected individuals [50, 98] and a cell-mediated immune response is also involved [100].

A previous study demonstrated that intranasal immunization with the 62-kDa proteinase of *T. vaginalis* with adjuvant confers protection in mice, suggesting that the levels of IgA are important in protective immune responses against *T. vaginalis* [47].

In addition, designing cysteine proteinase inhibitors as drugs could be another contribution to the control of the infections [77], but requires the knowledge of which CPs are essential to the parasites. Protease inhibitors have generated interest as therapies and have proven to be of great value in the control of parasitic diseases, including malaria [85], trypanosomiasis [20], and angiostrongylosis [68]. Progress in this area for trichomonosis has been minimal, but advances in the characterization of parasite proteases could expedite new drug discovery efforts. Recently, the *T*. *vaginalis* protein phosphatase 1 gamma (TvPP1γ) has been considered a potential novel drug target for treatment of trichomonosis [73].

In most cases, understanding the role of trichomonal CPs has been limited by difficulties in obtaining enough quantity for protein purification and characterization, although recent advances have provided recombinant proteinase for more detailed study. Despite this, researchers have obtained important results that have led to a better knowledge of the parasite’s pathogenesis [81].

## Conclusions

It is evident that the pathogenicity of *T. vaginalis* is multifaceted. Despite the frequency of infection by *T. vaginalis*, basic components of the disease process are still unknown. Cysteine proteinases are key proteins in the metabolic process; the knowledge of the roles of some CPs in the onset of the infection are very important; it will be useful in order to develop targeted intervention strategies such as vaccines and drugs. A *T. vaginalis* vaccine and the identification of promising targets for drug development could provide short-term cures, reduce medical costs, and prevent sequel associated with pregnancy and infertility. More research is needed to improve our understanding of this parasite infection.
